# The central role of neutrophil extracellular traps (NETs) and by‐products in COVID‐19 related pulmonary thrombosis

**DOI:** 10.1002/iid3.949

**Published:** 2023-08-28

**Authors:** Shi Li, Hui Wang, Qixiang Shao

**Affiliations:** ^1^ Department of Immunology School of Medicine, Jiangsu University Zhenjiang Jiangsu China; ^2^ Department of Medical Microbiology and Immunology, Institute of Medical Genetics and Reproductive Immunity, School of Medical Science and Laboratory Medicine Jiangsu College of Nursing Huai'an Jiangsu China

**Keywords:** COVID‐19, inflammation, neutrophil extracellular trap, neutrophils, pulmonary embolism

## Abstract

Extracellular trap networks (neutrophil extracellular traps [NETs]) of polymorphonuclear neutrophils are mesh‐like substances that prevent the spread of pathogens. They primarily consist of DNA skeletons, histones, granule components, and cytoplasmic proteins. NETs formation requires a certain environment and there are different pathways for NETs production. However, it is still not clear how severe acute respiratory syndrome coronavirus 2 (SARS‐CoV‐2) promotes NETs. NETs exert antiinflammatory effects through immune response, while they can also lead to certain adverse outcomes, such as the development of immunothrombosis. Coronavirus disease 2019 (COVID‐19) is an inflammatory reaction affecting various organs caused by SARS‐CoV‐2, especially the lungs. NETs production and disease severity are linked with unique neutrophil clusters by single‐cell RNA sequencing. NETs might exert an anti‐inflammatory role in the initial stage of lung tissue inflammation. Nevertheless, numerous studies and cases have shown that they can also result in pulmonary thrombosis. There is mounting evidence that NETs are tightly related with COVID‐19 pulmonary thrombosis, and many studies on the mechanisms are involved. The role and mechanism of NETs in the development of pulmonary thrombosis will be the main topics of this manuscript. Additionally, we address the potential targeting of NETs in COVID‐19 patients.

## Graphical abstract

The respiratory tract is primarily infected by SARS‐CoV‐2. The primary cause of mortality for COVID‐19 was acute respiratory distress syndrome (ARDS), which can be brought on by a lung infection. This review we mainly discuss the mechanism that favor NETosis in COVID‐19 immune pulmonary thrombosis, as well as the potential targeting of NETs in COVID‐19 patients.

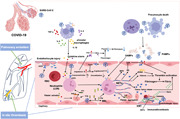

## 1 INTRODUCTION

The sudden outbreak of the severe acute respiratory syndrome coronavirus 2 (SARS‐CoV‐2) pandemic in 2019 has caused a major global impact. According to the latest statistics released by Johns Hopkins University (JHU) on March 11, 2023, the cumulative number of confirmed cases of the coronavirus disease 2019 (COVID‐19) in worldwide reached 676,609,955, with a total death toll of 6,881,955. SARS‐CoV‐2 is distinct from prior human perceptions of the virus. It rapidly mutates to become highly resistant to the outside world and is extremely contagious, repeatedly eluding the immune system and causing extensive transmission.^1^ Additionally, COVID‐19 has also been linked to numerous complications, most notably severe pulmonary thrombosis.^2^ The mechanisms, nevertheless, are still not completely elucidated. Numerous researches have demonstrated that neutrophil extracellular traps (NETs) are closely associated with inflammatory responses as well as pulmonary thrombosis. Moreover, recent studies have confirmed the association of NETs with the development of COVID‐19 pulmonary embolism (PE).^3^, ^4^, ^5^ In this review, the formation mechanism of NETs, and their contribution to the development of pulmonary thrombosis in COVID‐19 and its treatment are summarized. Furthermore, the mechanism of pulmonary thrombosis of COVID‐19 will be addressed from a new perspective and considered for the clinical diagnosis and management of inflammatory thrombosis.

## 2 NETs FORMATION AND ITS ROLE IN THE SARS‐COV‐2 INFECTION

### 2.1 The process of formation of NETs and NETosis

The formation of NETs is a complicated process. Numerously experimental studies have elucidated that common pathogens such as bacteria, fungi, protozoa and viruses can induce the activation of polymorphonuclear neutrophils (PMNs) and promote the formation of NETs.^6^ In addition, NETs can be produced in vivo by activated platelets, LPS, autoantibodies, granular antigen‐antibody complexes, and cytokines, such as interleukin‐8 (IL‐8), G‐CSF, tumor necrosis factor (TNF), interferon‐γ (IFN‐γ), and transforming growth factor‐β (TGF‐β).^7^ Treatment with phorbol myristate acetate (PMA) and Ionomycin in vitro similarly increased NETs formation.^7^ It has been found that a variety of receptors, humoral immune molecules, and signaling transduction pathway are necessary for the formation of NETs, such as G protein‐coupled receptors, TLR‐2, complement C3, TNF, FcR and IL‐1R/TLR, and their downstream signals. Some reports also demonstrated that CD18 is required for the formation of NETs which are induced by platelets activation, *staphylococcus aureus* (*S. aureus*) and hantavirus.^8^ Reactive oxygen species (ROS) production, transport of neutrophil elastase (NE) and myeloperoxidase (MPO) from neutral granules to the nucleus, and histone modifications leading to DNA dissociation are the four stages involved in NETs formation. Fuchs and his colleagues found that PMNs were immediately activated using live‐cell imaging in response to risk factors. Upon stimulation, PMNs changed from spherical cells to flattened ones, and the cytoplasm formed a foamy shape and attached to the exterior matrix. The nuclear membrane remained intact an hour later, but the typical lobed nuclei gradually disappeared and chromosomes started to depolymerize. At last, the nuclear membrane was shed, and the cell membrane ruptured to release NETs out of the cells, in which PMNs trapped and killed pathogens. It was accompanied by the death of PMNs in a non‐apoptotic and non‐necrotic manner.^9^ In 2004, Brinkmann discovered and named NETs using high‐resolution transmission electron microscopy (TEM) and scanning electron microscopy (SEM), and also analyzed the components of NETs. This inflammatory death pattern associated with PMNs during the formation of NETs was named NETosis.^10^

### 2.2 Structure and composition of NETs

NETs are composed of smooth fiber with 15–17 nm in diameter and spherical structures with around 25 nm in diameter. These spherical structures can be aggregated into a spherical zone with a diameter of 50 nm and a hole about 30 nm in the middle.^10^ The structure allows a high density of sterilization space to be generated in a specific area.^10^, ^11^ The nuclear histones, histone G (Cathepsin G, CG) in PMNs, NE and MPO, and other granular proteins such as S100 calcium‐binding proteins A8, A9, and A12, actin and α‐actin are embedded on the depolymerized chromatin that makes up the skeleton of NETs. The DNA skeleton is an essential component of NETs because once the DNA skeleton of NETs is disrupted by DNase, PMNs lose their extracellular bactericidal effect.

### 2.3 Function and mechanism of NETs

There are two fundamentally different types of NETs formation and NETosis. ① Lytic/suicidal NETosis frequently occurs as a result of danger signals such as PMA, uric acid crystals and pathogens. When receptors on PMNs bind to ligands, the downstream Raf‐MEK‐ERK pathway is activated, and the cells undergo a respiratory burst that activates NADPH oxidase and causes the generation of ROS such as O_2_ and H_2_O_2_.^12^, ^13^ ROS participates in the production of NETs in addition to its capability to kill bacteria. The formation of NETs is dependent on autophagy and ROS generation, as Remijsen discovered.^14^ Recent studies have shown that PAD4 is activated by both ROS‐dependent and nondependent pathways through receptor interacting protein kinase‐1/3 (RIPK1), which in turn activates caspase‐8 or mixed lineage kinase domain‐like protein (MLKL) cascade reaction to cause the formation of NETs.^15^, ^16^ Moreover, gasdermin D (GSDMD) has been found recently to mediate NETosis. The GSDMD complex perforates the PMNs granular membrane, releasing NE and MPO. Furthermore, NE and GSDMD formed a positive feedback loop. ② Vital NETosis, a rapid process of NETs formation, is usually induced mainly by certain pathogens binding to TLRs on the cell surface. This process is independent of NADPH, which accounts for about 20% of NETosis. The depolymerized DNA, wrapped in a nuclear membrane, forms vesicles that go through the cytoplasm, and excrete DNA in the way of exocytosis. Therefore, vital NETosis does not result in the destruction of the cell membrane, while nonnucleated PMNs are still able to undergo chemotaxis and phagocytosis of the pathogen. The rapid generation of NETs is also an important mechanism of anti‐infection, and its role is mainly in the early stages of infection.

### 2.4 NETs in SARS‐CoV‐2 infection

SARS‐CoV‐2 were found in neutrophils of the blood samples from the COVID‐19 patients. It has been demonstrated that SARS‐CoV‐2 has the ability to encourage NETs release by human neutrophils directly in vitro. The ACE2‐TMPRSS2 axis is essential for SARS‐CoV‐2 entrance and neutrophil NETs release.^17^ SARS‐CoV‐2, on the other hand, can infect host cells via noncanonical receptors such as C‐type lectin receptors (DC‐SIGN and L‐SIGN) and sialic acid‐binding immunoglobulin‐like lectin 1 (SIGLEC1), which have recently been shown to enhance NETs formation in dengue fever.^18^, ^19^ Platelets robust aggregated SARS‐CoV‐2‐induced NETs formation, CLEC5A and TLR2 inhibition may help to alleviate lung inflammation thrombosis in COVID‐19 patients.^20^ SARS‐CoV‐2 activates pathogen recognition receptor signaling, resulting in the simultaneous release of IFNs and other proinflammatory cytokines.^21^ Metabolites analysis revealed that neutrophils from SARS‐CoV‐2 patients have shown higher ROS emission. Also, COVID‐19 patients have higher levels of glutathione synthesis, which is probably a defense mechanism against NETs formation.^22^

Major changes are brought about in the neutrophil compartment by SARS‐CoV‐2 infection. Low‐density neutrophils (LDNs), a mixture of both mature and immature neutrophils, have been demonstrated to be more susceptible to NETosis and NETs formation.^23^ Patients with severe COVID‐19 have greater upregulation of LDG levels, which is a sign of a poor clinical prognosis. These immature neutrophils with phenotypic markers of immunosuppression and malfunction.^24^ Furthermore, analysis of COVID‐19 whole blood samples using single‐cell RNA sequencing identified unique neutrophil clusters linked with NETs production and disease severity.^25^

### 2.5 Host defense and proinflammation response of NETs in SARS‐CoV‐2 infection

PMNs play an important role in the innate immune response, which is the human immune system's first line of defense. After infection, PMNs phagocytose pathogens, subsequently degranulate, and chemotactic and activate other immune cells by secreting cytokines. When chemoattractant substances are released from local infection sites, PMNs can enter the tissues of most organs from the circulation, especially in highly vascularized organs such as the lung, which is the main target organ of COVID‐19.^26^ The anti‐infective effect of NETs is mainly through the trapping and direct killing of pathogens.^27^ NETs first produce a high concentration of bactericidal environment locally to inactivate pathogens. After that, NE in the NETs of PMNs has a unique ability to degrade and kill pathogens. In addition, other antimicrobial peptides, proteins (lysozyme, protease) and ion chelating agents have bactericidal effects.^28^ It is of interest that histones, which are part of NETs, can also kill pathogens at low concentrations. Moreover, NETs act as a physical barrier to limite spread of the pathogens and the release of inflammatory mediators, preventing harm to normal tissues.^10^ Transcriptome results of COVID‐19 patients revealed 16 NETs‐associated genes connected with the innate immune response (via IFN signaling) and T, B, and NK cells.^29^ However, NETs have been shown to have both pathogen‐suppressive and inflammation‐promoting effects, making them a double‐edged sword. They promote inflammation,^30^ adaptive immune response,^31^ endothelial injury^32^ and pulmonary thrombosis^33^ by inducing type I interferon (IFN‐Ⅰ) and the formation of Nod‐like receptor thermal protein domain associated protein 3 inflammasome induced by inflammatory cytokines. Precision control of IFN‐Ⅰ response is critical since both overactivation and under activation of IFN signaling can be detrimental to the host. Clinical reports show that severe COVID‐19 cases result in high levels of cytokines such as IL‐6, IL‐8, TNF, and G‐CSF. All of these compounds have the ability to strongly activate neutrophils. As a result, a proinflammatory environment contributes to the quantity and effector function of neutrophils in COVID‐19.^34^ NETs can promote the activation of platelets and help the aggregation of activated platelets and red blood cells, which plays an important role in the formation of inflammatory thrombosis caused by NETs.^35^ In addition to bactericidal effects, NE on NETs can also inactivate tissue factor (TF) pathway inhibitors, thereby breaking the balance between coagulation and anticoagulation and promoting the formation of inflammatory thrombosis.^36^ These mechanisms are closely related to the formation of pulmonary thrombosis in COVID‐19 patients. The presence of NETs has been found in a variety of different organs, thus causing multiorgan damage. This is also an important cause of multiple organ dysfunction syndrome in COVID‐19 patients.^37^ Indeed, in many types of inflammation, interfering with the release or inducing the clearance of NETs can prevent tissue damage. For instance, NETs‐binding protein aggregation can accelerate NETs capture and cut off inflammatory mediators, attenuate inflammatory responses, and alleviate inflammation.^38^

### 2.6 Techniques for visualization and quantification of NETs

Brinkmann and colleagues first discovered NETs using TEM and SEM. The SEM revealed the structure of NETs, whereas TEM allowed for the assessment of the morphology of NETs‐releasing cells.^10^ Recent research suggests that thrombosis in COVID‐19 patients is connected to increased NETs production. Numerous studies have assessed higher amounts of NE, histone‐DNA, MPO‐DNA, NE‐DNA, circulating cell‐free DNA (cfDNA), and (citrullinated) histone H3 (H3cit) in blood, plasma, and tissues, and higher levels of NETs formation were found to be connected with disease severity.^39^ Immunohistochemistry can identify NETs components in tissues. A significant development in the study of NETs formation was the intravital microscopy's real‐time capture of a NETs‐forming cell in vivo.^40^ However, it can be challenging to obtain tissue samples, blood and plasma samples are commonly identified by enzyme‐linked immunosorbent assay. In addition, MPO/H3cit positive neutrophils and serum dsDNA were detected by flow cytometry to measure cellular and extracellular NETs.^40^

Compared to healthy controls, blood neutrophils from COVID‐19 patients secreted more NETs, MPO‐DNA complexes were found in higher concentrations in the plasma of COVID‐19 patients.^41^ Furthermore, compared to neutrophils from healthy controls, COVID‐19 patients' neutrophils released more NETs from the branches. When extracellular DNA was stained with MPO and H3Cit, the confocal microscopy analysis of tracheal aspirate pellet cells and lung tissue from autopsies revealed typical NETs structures as well.^17^ Also, platelet accumulation and microvascular thrombosis in the lungs are frequently found in post‐mortem examinations.^42^

## 3 PULMONARY THROMBOSIS ASSOCIATED WITH COVID‐19

### 3.1 Occurrence of severe COVID‐19 and its association with pulmonary thrombosis

While most COVID‐19 patients only experience moderate cold symptoms such as fever, coughing, and a decreased sense of smell and taste, a few patients may experience severe clinical symptoms and even die.^43^ Severe COVID‐19 is closely related to other underlying illnesses such as diabetes, hypertension, and cardiovascular disease.^44^ Approximately 10% of COVID‐19 patients can suffer from life‐threatening multiorgan failure, which puts unprecedented pressure on the healthcare system.^45^ Understanding the pathogenesis of severe COVID‐19 is crucial for the effective treatment of COVID‐19 patients. A large amount of evidence has confirmed that the pathogenesis of severe COVID‐19 is closely related to microthrombosis and coagulation disorders.^46^ Patients with severe COVID‐19 symptoms typically have higher D‐dimer and fibrinogen degradation products.^47^ While patients with coagulation disorders also have a poor prognosis, with a mortality rate of nearly 40%.^48^ These phenomena suggest that coagulation disorders are a high‐risk factor for COVID‐19. The rapid response of the patient's innate immune system following SARS‐CoV‐2 infection and the direct interaction of damaged alveolar epithelial cells, activated leukocytes, platelets and plasma coagulation factors, which in turn cause thrombotic events in patients with COVID‐19, are potential mechanisms.^49^ Although thrombosis existed in different organs such as the heart, kidney and brain, inflammatory thrombosis is more pronounced in the lungs. SARS‐CoV‐2 primarily infects the respiratory tract. The lung infection can cause acute respiratory distress syndrome (ARDS), which is the main reason of death for COVID‐19. The lungs exhibit the largest pathologic damage, as seen by alveolar destruction, inflammation, and thrombosis.^50^, ^51^, ^52^ It is well documented that SARS‐CoV‐2 infection increases the risk of venous thromboembolism (VTE), deep vein thrombosis (DVT) and PE make up the majority of VTE cases. However, many studies did not distinguish between DVT and PE. The investigation from autopsies performed on 45 patients with SARS‐CoV‐2 infection found that most samples were regarded as primary thrombi as there was no information indicating DVT. Histologically, numerous microthrombi in capillaries and larger main thrombi in arterioles were seen in 87% of the samples. Only 9% of deaths were linked to clinically confirmed DVT and/or thrombosis of the right heart chamber.^50^ Another research summarized 27 studies including 3342 patients with COVID‐19, more than half of patients with PE lacked DVT.^46^ Data from case reports indicate a large variation in the interval between COVID‐19 diagnosis and PE happened, which requires more attention for clinical management. In addition, the high incidence of VTE in COVID‐19 patients indicates that different mechanisms outside the known ones are involved in COVID‐19 associated pulmonary thrombotic events.^53^ Further, extracellular vehicles were found to contain SARS‐CoV‐2 RNA according to Barberis et al.^54^ Through the circulatory system, the SARS‐CoV‐2 RNA can be concealed, transferred, released, and re‐attack other tissues and organs. These could be possible explanations for the continued thrombotic risk in COVID‐19 individuals.

### 3.2 Immune disorders and thrombosis mechanisms in COVID‐19 patients

It has been widely reported that COVID‐19 patients have immune dysfunction. A severe immune dysfunction arising from SARS‐CoV‐2 infection is manifested as the cytokine storm syndrome, also known as cytokine release syndrome.^55^ After SARS‐CoV‐2 infection, innate immune cells such as monocytes, macrophages (Mφ), dendritic cells (DCs) and NK cells secrete large amounts of various cytokines, including IL‐6, IL‐1, GM‐CSF, IFN‐α, TNF‐α, chemokines and their ligands.^56^ These cytokines facilitate the activation of immune cells, immune response and further chemotaxis of other immune cells to eradicate infections. Although excessive cytokines can boost immune response, they can also result in excessive local inflammation, which leads to nearby cell and tissue damage, cause apoptosis of lung epithelial and endothelial cells, activate the coagulation system, promote thrombosis, and even cause multiorgan failure.

In 2020, Zhang^57^ and his colleagues first reported that SARS‐CoV‐2 infection caused a significant increase in multiple antiphospholipid antibodies (aPL antibodies) in patients, suggesting an association with coagulation abnormalities and pulmonary thrombosis in patients with COVID‐19. It has been shown that elevated aPL antibodies can be caused by autoimmune responses or viral infections. The aPL antibodies that are positive in COVID‐19 patients indicate impaired immune function. The blood of COVID‐19 patients is hypercoagulable due to several factors, and the presence of aPL antibodies will increase the risk of clotting. It may not only aggravate the acute clinical symptoms of patients, but also raise the likelihood of developing chronic autoimmune diseases. Zuo et al.^58^ determined the aPL antibodies and verified IgG isolated from COVID‐19 patients. NETs can be dissolved by micrococcal nucleases for MPO, and NETs can be measured by detecting their enzyme activity. The findings demonstrate that IgG components of aPL antibodies from sera of COVID‐19 patients promote NETs release.^58^ Increased adhesion potential in some PMNs with antiphospholipid syndrome is linked to the activation of the integrin Mac‐1. This adhesion characteristic lowers the threshold for thrombosis, promotes endothelium‐neutrophilic interaction, and facilitates the formation of NETs.^59^ Many studies have shown that lowering the formation of NETs can lower the risk of thrombotic disease. For example, aPL antibody‐mediated NETosis formation can be prevented by agonism of the adenosine A2A receptors via the protein kinase A pathway.^60^ In clinical practice, the hypercoagulable state of COVID‐19 patients should receive extra attention and symptomatic treatment with appropriate anticoagulants and antiplatelet medicines, and if necessary, hormone therapy to suppress the autoimmune response^61^ to lower the risk of thrombosis and prevent the development of severe disease.

The renin‐angiotensin system has been reported that plays an important role in the development of COVID‐19.^62^ SARS‐CoV‐2 was identified as β coronavirus that can invade cells through its surface spike protein (S protein) combined with cell surface angiotensin converting enzyme 2 (ACE2).^63^ ACE2 is widely expressed in heart, lung, kidney and adrenal tissues, highly expressed in endothelial cells, which is closely related to the multiorgan vascular endothelial injury caused by SARS‐CoV‐2 and the formation of thrombus. Endothelial cells have limited procoagulant activity under physiological conditions. Under the infection of SARS‐CoV‐2, endothelial cells are stimulated by cytokines and NETs, augmenting the expression of TF, thereby promoting the occurrence of coagulation cascade.^64^ Patients with high ACE2 expression are more prone to develop serious illness.^62^ Meanwhile, angiotensin II (Ang Ⅱ) can promote vasoconstriction, inflammatory response and thrombosis by binding to its type 1 receptor (Angiotensin Ⅱ 1 receptor, AT1R) and type 2 receptor (AT2R). ACE2 can also contribute to the conversion of Ang Ⅱ to Ang‐(1‐7), which binds to the G protein coupled Mas receptor (MasR) and activates signaling pathways to counteract the effect of the Ang II/AT1R pathway and play the role of vascular dilatation. Ang‐(1‐7) is a kind of substance with strong anticoagulant activity, and the mechanism underlying this process may be related to NO and PGI2.^65^ Under the physiological state, Ang II and Ang‐(1‐7) are balanced by each other. As SARS‐CoV‐2 infection leads to ACE2 internalization, the balance shifts to Ang II‐mediated vasoconstriction and thrombosis, and promotes pulmonary thrombosis.

In addition, PMNs‐derived TFs and NETs play important roles in the formation of pulmonary thrombosis during COVID‐19. The aggregation and activation of PMNs on the endothelial surface of blood vessels is the initiating process that causes thrombosis, and a high concentration of TF within NETs plays a pivotal role in the beginning and progression of thrombosis. Previous studies have found that many inflammatory cells exude from the vessel wall at the early stage of thrombosis, and TF is increased after thrombosis.^66^ TF is a transmembrane glycoprotein that is an exogenous coagulation initiator and a member of the human class II cytokine receptor family.^30^ It plays a critical role in the exogenous coagulation pathway. TF has a high affinity with factor Ⅶ, which can be combined with each other to form a complex that promotes the activation of FⅦ to FⅦa. In addition, TF can enhance the activity of FⅦa and TF/FⅦa protein hydrolase, which leads to the activation of factor X. After that, the activated factor X can encourage the conversion of prothrombin into thrombin and fibrinogen into fibrin by the action of thrombin, thus promoting thrombus formation. This view was supported by Mackman's findings as well. They found that TF is closely related to the activation of a series of coagulation factors in the exogenous coagulation process, and a large amount of TF would be secreted in vascular endothelial cells and blood during the inflammatory response, which triggers the formation of vascular and venous thrombi.^67^ Increased expression of plasminogen activator inhibitor‐1 (PAI‐1) in the mononuclear macrophage system and decreased antithrombin in patients with COVID‐19 further promote the occurrence of blood coagulation.^68^ Clinical studies have shown that patients with inflammatory diseases are more like to develop venous thrombosis. If TF is relatively insufficient or protease activated receptor 2 (PAR2) is deficient, the inflammatory process can be relieved.^69^ Furthermore, exogenous coagulation cascade inhibitors can also attenuate or block the progression of inflammation.^70^ These findings offer a solid foundation for exploring new treatments for COVID‐19 patients who have PE.

## 4 MECHANISM OF NETs CONTRIBUTE TO PULMONARY THROMBOSIS DURING COVID‐19

### 4.1 Experimental evidence for the role of NETs and their degradation products in thrombosis

Numerous studies suggested that NETs play an important role in the development of thrombosis. Darbousset and his colleagues found that when endothelial cells are damaged, PMNs infiltrate and induces thrombus formation.^71^ Clark also demonstrated that intravascular NETs in mice with endotoxin‐related sepsis boosted platelet aggregation and activation, thereby damaging endothelial cells, altering hemodynamics, and promoting thrombosis.^35^, ^72^ Fuchs' results verified that NETs promoted thrombosis and served as a scaffold for platelet adhesion, aggregation and activation.^73^ Brill further confirmed the scaffolding role of NETs in DVT, and fibrin and Von‐Willebrand factor as thrombosis scaffolds. Although NETs can aid in the clotting response and limit the spread of pathogenic bacteria, excessive activation of NETs can lead to massive thrombus formation and cause severe tissue ischemia.^27^ In contrast, heparin promotes the dissociation of NETs, thus inhibiting thrombosis caused by the direct activity of NETs.^74^ However, it has also been shown that NETs themselves play a weak role in promoting coagulation, but their degradation products, histones and DNA, can promote thrombosis through different mechanisms of action. Histones bind to thrombomodulin (TM) and protein C, and increase plasma thrombin production by inhibiting TM‐dependent protein C activation, leading to the conversion of fibrinogen to insoluble fibrin, and thus microthrombus formation.^75^ Negatively charged DNA is also a potent procoagulant and may be associated with promoting the activation of certain coagulation factors.^7^ However, heparin is unable to inhibit the procoagulant effect of NETs cleavage products.^76^

### 4.2 The role of PMNs and NETs in COVID‐19 immune pulmonary thrombosis

NETs are involved in thrombosis, and numerous studies have shown that NETs can release large amounts of TF, and can co‐localize with TFs and von Willebrand factors to initiate the exogenous coagulation system and activate the endogenous coagulation pathway via Ⅻ‐FⅫ, trap and activate platelets.^77^ NETs also play an important role in the maintenance of intravascular thrombosis. PMNs infiltration, degranulation, and NETs release in late COVID‐19 can lead to severe inflammatory responses and cardiovascular impairment. Whereas, in patients with severe COVID‐19, NETs degradation products containing cfDNA, guanosine histone H3, DNA‐NE and DNA‐MPO complexes are widespread in the circulation^78^, ^79^ and promote thrombin activation,^75^ which in turn promotes the development of hemagglutination.

In addition, in COVID‐19, SARS‐CoV‐2 activates complement through interaction with mannan‐binding lectins, serine proteases (MASPs), or autoantibodies or/and immune complexes, leading to the production of C3a, C5a, and sC5b‐9. Platelets are activated by C3a, which releases neutrophil activating peptide‐2(NAP‐2) and platelet factor 4(PF4), which further stimulate neutrophil and monocyte/macrophage activation.^80^ Platelets also adhere to damaged lung capillaries. They produce adhesion molecules upon activation, which induce the aggregation of PMNs.^37^ While C5a and activated platelet‐derived thrombin induce elevated TF expression in PMNs and release NETs that are loaded with activated TF. NETs induce TF expression and activation of endothelial cells, thereby increasing their procoagulant activity, further amplifying inflammation and platelet activation, thus feeding the vicious cycle of complement/NETs‐driven immunothrombosis.^81^ These results demonstrate that the development of hypercoagulation and thrombosis in COVID‐19 patients is due to the interactive activation of NETosis, the coagulation system and the complement pathway, forming a positive feedback loop. The overproduction of NETs also causes direct vascular damage and indirectly promotes the production of autoantibodies, which leads to several types of autoimmune vasculitis.^82^, ^83^ Furthermore, NETs can also directly activate vascular endothelial cells and induce epithelial‐to‐mesenchymal transition and apoptosis. SARS‐CoV‐2 induces apoptosis of lung epithelial cells suggests that NETs have a disruptive effect on vascular endothelial integrity, barrier function, and lead to endothelial dysfunction.^84^, ^85^ NETs are present in the kidneys, lungs and circulation of COVID‐19 patients.^86^ The clinicopathological autopsy also confirms that large amounts of aggregate NETs in the lung tissues of COVID‐19 patients with ARDS cause extensive PE.^79^ Therefore, the accumulation of NETs can lead to multiple organ failure in addition to pulmonary thrombosis.^87^ These findings imply that complement, thrombin and NETosis are promising therapeutic targets for pulmonary thrombosis in patients with COVID‐19.

**Figure 1:**
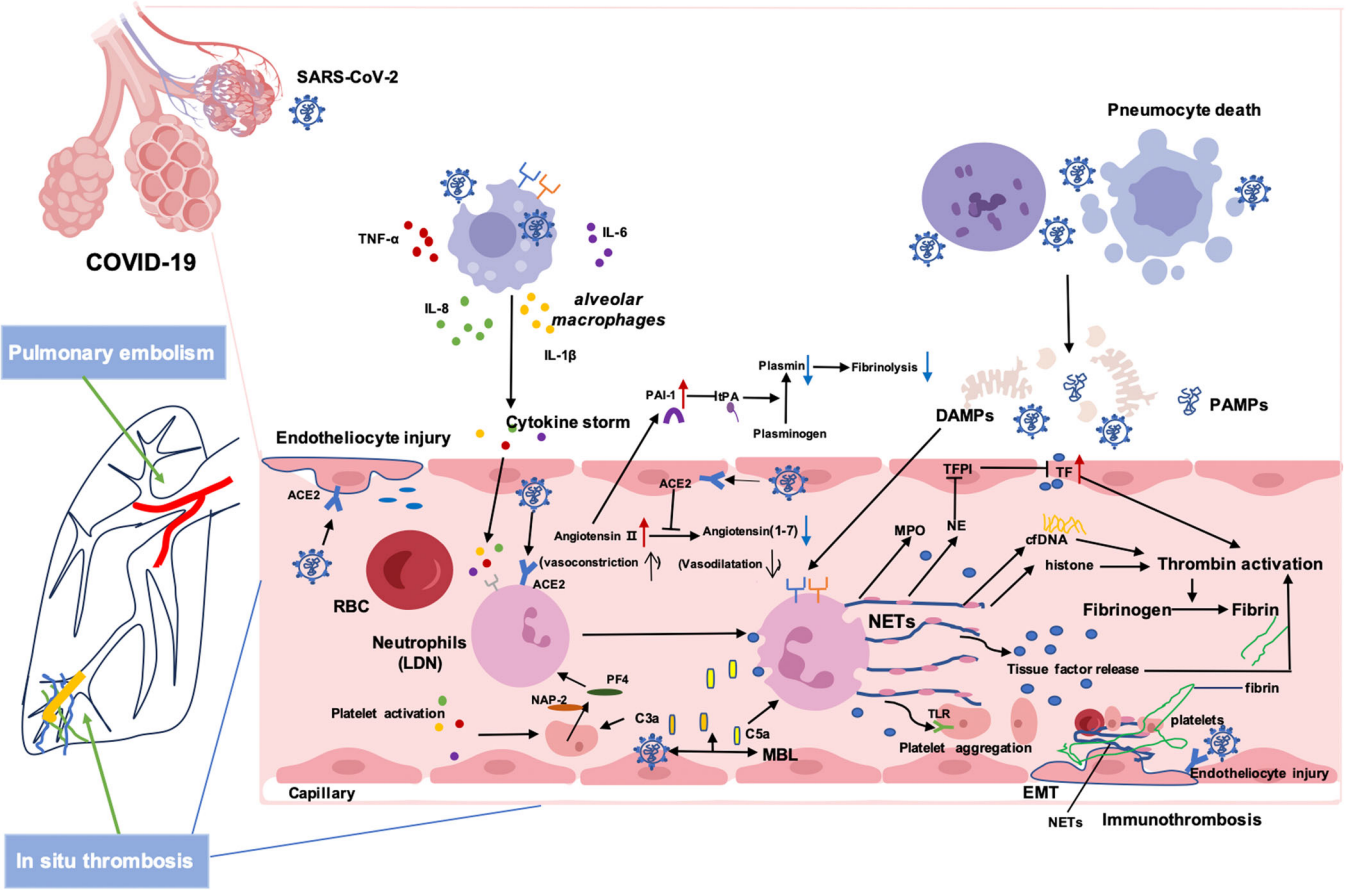
The mechanism of NETs in pulmonary thrombosis of COVID‐19. COVID‐19, coronavirus disease 2019; NETs, neutrophil extracellular traps.

Increasing evidences show that alveolar macrophages (AMs) can be impacted by SARS‐CoV‐2. It induces AMs to activate and differentiate into the “M1” phenotype, which can produce a large amount of TNF‐α, IL‐1β, IL‐6, IL‐8, chemokines and other inflammatory factors.^88^ Inflammatory cytokines enter the blood vessels of the lungs, setting off a chain of events that activates vascular endothelial cells,^89^ platelets^90^ and PMNs,^91^ and finally forming platelet‐PMNs complex on the endothelial surface,^92^ thus promoting the accumulation of PMNs and inflammatory activated platelets into the alveolus and lungs, and causing ARDS. At the same time, NETs are released and cell death is induced when PMNs infiltrate into the alveoli and interstitial of lung. This caused severe tissue damage, pulmonary impairment and hypercoagulability, which is typically characterized by an overactive coagulation cascade and a relatively depleted fibrinolytic system. It promotes the occurrence of diffuse intravascular coagulation (DIC), leading to local ischemia and hypoxia, tissue damage, and the release of endogenous damage‐related molecular patterns (DAMPs) such as HMGB1. Meanwhile, COVID‐19 patients infected with SARS‐CoV‐2 release large amounts of pathogens associated molecular patterns such as viral RNA. DAMPs, released from injured tissues, bind with corresponding receptors to activate PMNs, macrophages and other innate immune cells, generate NETs and the activate TF, and cause endogenous and exogenous intravascular coagulation activation (Figure 1). The interaction between activated innate immune cells and PMNs further speeds up the formation of thrombosis, a process called immunothrombosis,^89^ which thereby causes intravascular microthrombosis, pulmonary artery strangulation circulation, and is involved in the development of DIC and subsequent multiorgan damage or failure.^93^

The hypothesis of NETs in COVID‐19 associated pulmonary thrombosis. SARS‐CoV‐2 can activate LDNs and other cells to produce inflammatory cytokines and cause cytokine storms in moderate and severe COVID‐19 patients. The inflammatory environment promotes platelet activation, endothelial dysfunction, and complement activation. This inflammatory‐induced coagulopathy can culminate in a process known as immunothrombosis.

## 5 PROGRESSION IN NETs TARGETING THERAPY FOR PULMONARY THROMBOSIS IN COVID‐19 PATIENTS

The above studies provide a preliminary explanation of the role and mechanism of intrinsic immune cells, especially PMNs and their released NETs, in the development of pulmonary thrombosis and ARDS in patients with severe COVID‐19. This information is crucial for developing clinically individualized and precise therapeutic strategies that will improve the patients' survival rate. On the basis of the aforementioned findings, we contend that targeting NETs may similarly minimize thrombosis in patients with severe COVID‐19 (Table 1).

**Table 1:** Targeted therapies for NETs in COVID‐19.

Medication	Mechanism	PHASE	Patient population	NCT
DNase	Promoting the degradation of NETs	Ⅱ	Severe COVID‐19 pneumonia	NCT04541979
rh‐DNase	Promoting the degradation of NETs	Ⅱ	COVID‐19 patients with respiratory failure	NCT04445285
Anakinra	Inhibits NETs‐induced IL‐1‐mediated signaling	Ⅱ/Ⅲ	Severe COVID‐19 pneumonia	NCT04443881
Acetylsalicylic acid	Inhibiting platelet‐neutrophil interaction and reducing NETs formation	Ⅲ	Severe COVID‐19 pneumonia	NCT04808895
IVIG	eliminating NETosis	Ⅰ/Ⅱ	Severe and critical COVID‐19 pneumonia	NCT04521309
Sivelestat	inhibiting the formation of NE and NETs	Not Applicable	Acute respiratory distress syndrome due to COVID‐19	NCT05697016
Cyclosporine	inhibiting calcineurin pathway nuclear factor in activated T cells and NETs formation	Ⅳ	Severe COVID‐19 pneumonia	NCT04392531
C1 Inhibitor	Blocking the formation of histones, NETosis, and NETs	Ⅱ	Severe COVID‐19 pneumonia	NCT05010876
Metformin	Reduction of elastase, protease‐3, histone	Ⅱ	Severe COVID‐19 pneumonia and severe acute respiratory Syndrome secondary to SARS‐CoV‐2	NCT04625985

Abbreviations: COVID‐19, coronavirus disease 2019; IL, interleukin; NETs, neutrophil extracellular traps.

In recent years, NETs‐targeting drugs have drawn extensive attention. Medications have been developed targeting NETs in a number of ways. Inhibition of PAD4, NE, and GSDMD may block the development of NETs. Studies have shown that the PAD4 inhibitor Cl‐amidine can inhibit the generation of NETs and thus prevent the occurrence of thrombosis and lessen lung tissue damage by inhibiting the production of NETs, although it is currently in preclinical trials.^94^, ^95^ The production of depolymerized chromatin is prevented by a variety of PAD‐4 inhibitors with varying affinities, which is a necessary chromatin modification for the formation of NETs.^94^ It is well‐known that metformin can prompt insulin hypersensitivity. In addition, it can lessen the lung injury and pulmonary thrombosis caused by NETs by reducing the amount of NE, protease‐3, histone and double‐stranded DNA in NETs.^96^ Clinical trial using metformin in COVID‐19 are underway (Clinical Trials. gov identifiers: NCT04625985). Studies have shown that metformin is also beneficial in COVID‐19 diabetic patients.^97^

The NETs can be degraded by recombinant human DNase (rh‐DNase), which in turn lessens the inflammatory response.^98^ Patients with severe COVID‐19 had a large deficiency in the degradation of NETs in the serum, however, the additional administration of rh‐DNase enhanced the degradation of NETs.^99^ Additionally, it has been demonstrated that rh‐DNase treatment for COVID‐19 patients reduced the quantity of NETs found and improved the inflammatory condition of the patients' sputum, the patients' oxygenation function was additionally improved.^100^

Drugs that inhibit IL‐1 will stop an inflammatory circuit between NETs and IL‐1. Anakinra is a recombinant human IL‐1 receptor antagonist (rhIL‐1RA). Respiratory epithelial cells may secrete proinflammatory cytokines such as IL‐1α, IL‐8 and IL‐1β as a result of the release of NETs from COVID‐19 patients.^101^ IL‐1R agonists such as IL‐1α and IL‐1β can activate intracellular signaling pathways by binding to IL‐1 receptor 1 (IL‐1R1).^102^ Anakinra, Canakinumab (a humanized, monoclonal antibody against interleukin‐1β), and Rilonacept (another rhIL‐1RA) inhibit NETs‐induced IL‐1‐mediated signaling pathway.^101^ Anakinra has been test in Phase II and III trials with encouraging results (Clinical Trials. gov identifiers: NCT04443881). IL‐1β induces IL‐6 production, IL‐1β and IL‐6 inhibition may reduce NETs in severe COVID‐19 patients. Aspirin (acetylsalicylic acid) is a very widely used nonsteroidal anti‐inflammatory drug (NSAID) in clinical practice and is often used in a variety of inflammatory diseases.^103^ Aspirin acts as an antiplatelet agent by inhibiting cyclooxygenase (COX) and also inhibits the formation of NETs.^104^ The inhibitory effect of aspirin on NETs was also linked to its ability to block the nuclear factor kappa B pathway.^105^ Under inflammatory conditions, platelet activation by TLR4 signaling can promote the binding of platelets to PMNs, which can result in the formation of NETs.^72^ Given the interaction between PMNs and platelet cells, aspirin was found to block platelet‐neutrophil interaction and reduce NETs formation in pulmonary microcirculation and plasma PMNs.^106^, ^107^ Clinical trial is now being launched in Phase III (Clinical Trials. gov identifiers: NCT04808895).

Numerous autoimmune diseases are routinely treated with intravenous immunoglobulin (IVIG). IVIG has also been shown to reduce the formation of NETs.^108^ IVIG can eliminate NETosis in the presence of nigericin and PMA‐activated PMNs, as well as spontaneous NETosis in unstimulated PMNs.^109^

## 6 CONCLUSION

The mechanism of pulmonary thrombosis in patients with severe COVID‐19 is still being thoroughly studied, but it has been discovered that understanding the mechanism of NETs, complement and other mechanisms involved in pulmonary thrombosis can help with clinical diagnosis and treatment. The diagnosis and targeting for NETs in COVID‐19 are expected to make breakthroughs with the further elucidation of the mechanism of pulmonary thrombosis. Multi‐drugs such as rhIL‐1RA, monoclonal antibody against IL‐1, rh‐DNase, C1 Inhibitor and Aspirin, etc. may be the useful regents for the targeted therapy of embolism that caused by SARS‐CoV‐2.

## AUTHOR CONTRIBUTIONS

**Shi Li**: Conceptualization; writing—original draft. **Hui Wang**: Investigation; writing—review & editing. **Qixiang Shao**: Conceptualization; writing—review & editing.

## Data Availability

I confirm that I have included a citation for available data in my references section, unless my article type is exempt.
